# Supplementary material for: An unusual bird (Theropoda, Avialae) from the Early Cretaceous of Japan suggests complex evolutionary history of basal birds
Source: Commun Biol. 2019 Nov 14;2:399. doi: 10.1038/s42003-019-0639-4 (PMC6856171; doi:10.1038/s42003-019-0639-4)
Supplement: Supplementary file 1 — Supplementary Information [file 42003_2019_639_MOESM1_ESM.pdf]

Supplementary Figures

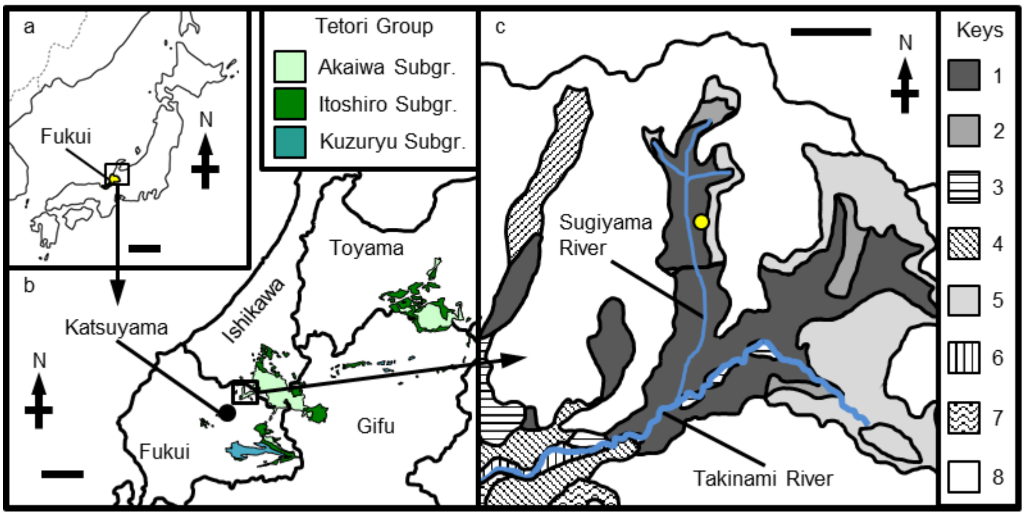

Supplementary Fig. 1 **a** Location map of Fukui, Japan. **b** Location map of Katsuyama and the surrounding area with distribution of the Tetori Group. **c** Geological map around the Kitadani Dinosaur Quarry (yellow circle), after ref. 1. Keys: 1, Kitadani Formation and underlying Akaiwa Formation, Akaiwa Subgroup, Tetori Group; 2, Omichidani Formation; 3, Hida Metamorphic Rock; 4, terrace deposits; 5, Nohi Rhyolite; 6, Quaternary alluvial deposits; 7, Quaternary fan delta deposits; 8, andesitic rocks. Scale bars equal 200 km in a, 20 km in b, and 4 km in c.

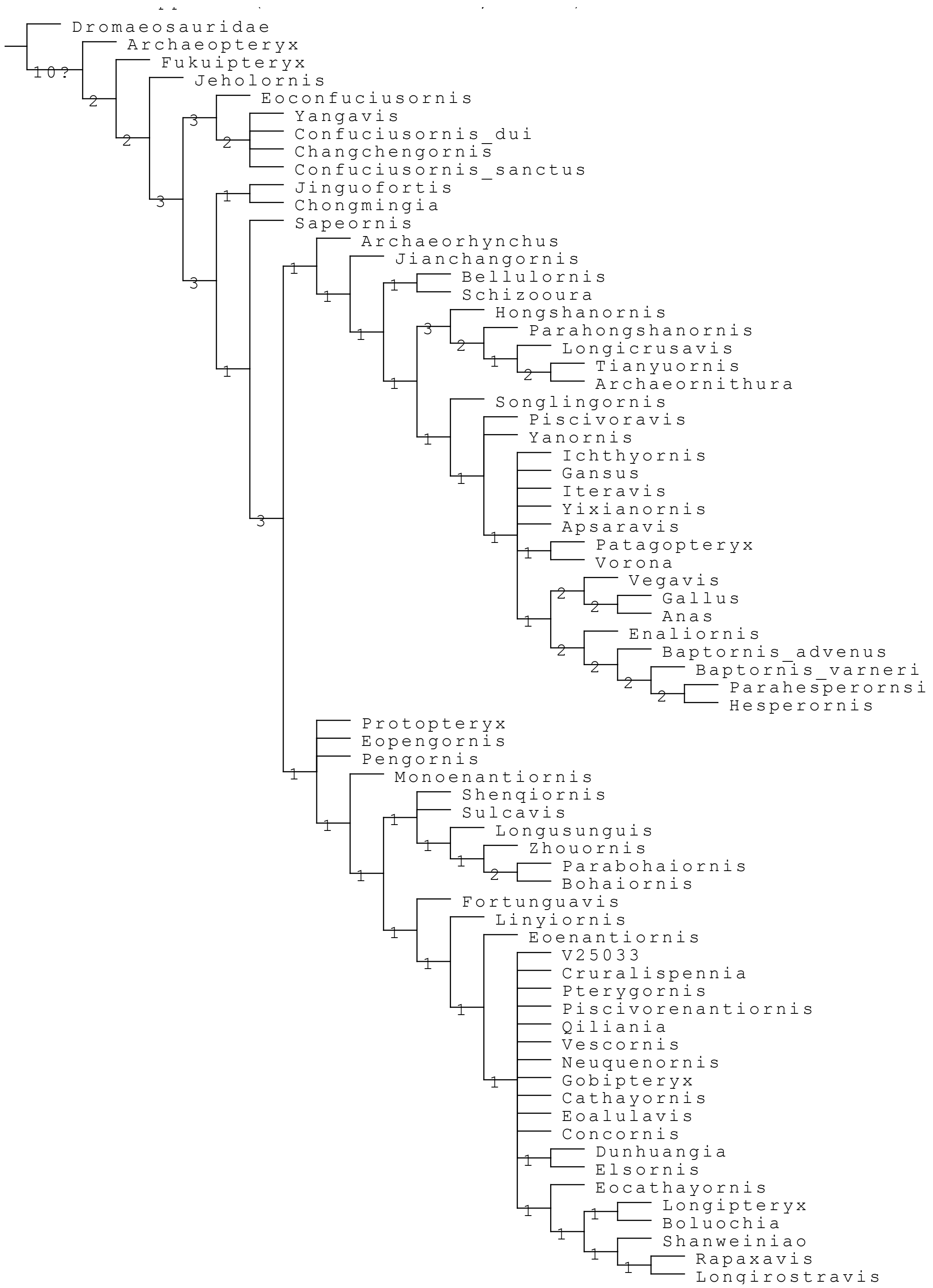

Supplementary Fig. 2 The strict consensus tree of 1344 most parsimonious trees with a length of 1323, resulting from analysis with addition of FPDM-V-9769 (*Fukuipteryx prima*) to a matrix on fossil birds[2]. Numbers near each node denote bremer support values.

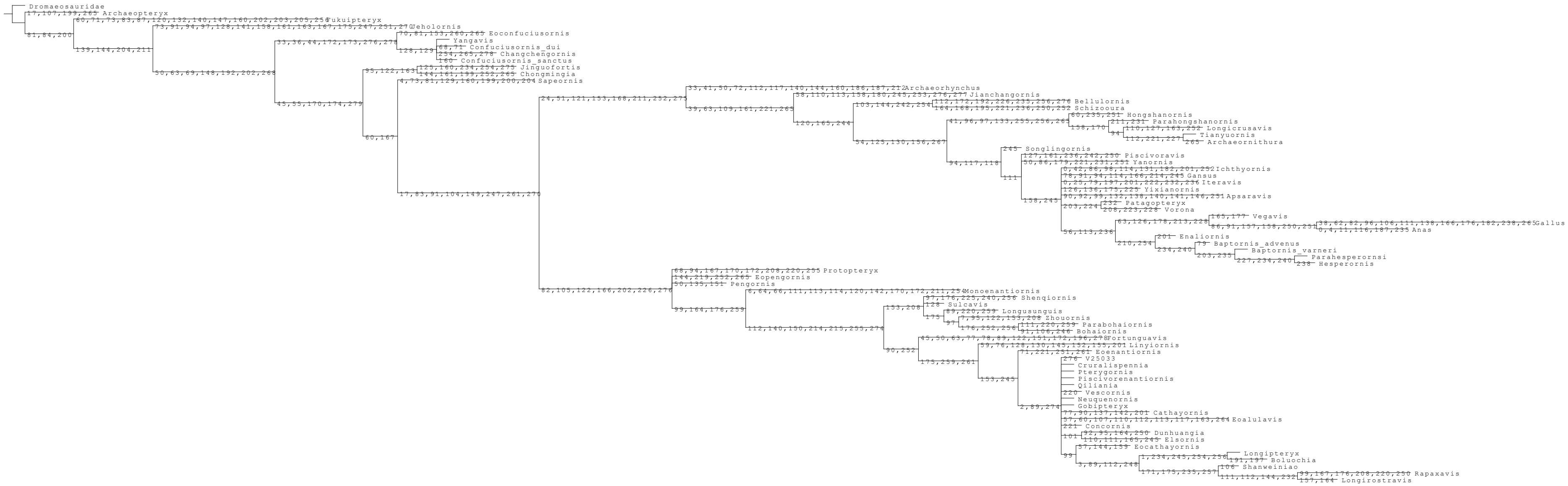

Supplementary Fig. 3 The strict consensus tree from Fig. S2 with common synapomorphies plotted for each avialan clade. The numbers on branches correspond to the characters in Supplementary Methods. Note that the default program for TNT expresses Character 1 in Supplementary Methods as 0, Character 2 as 1, Character 3 as 2… and so on in the above tree.

Supplementary Table

Elements	*Fukuipteryx* FPDM-V-9769 This Study	*Archaeopteryx* WDC-CSG-100 ref. 3	*Jeholornis* IVPP V13353 ref. 4	*Confuciusornis* IVPP V13156 ref. 5	*Jinguofortis* IVPP V24194 ref. 2	*Chongmingia* STM9-9 ref. 4	*Sapeornis* IVPP V13276 ref. 6
Py	26	-	-	30.5	23.89	?	13.7
Hum	75	57.8	88.6	61.3	66.64	72.6	121.5
Uln	71	51.6	90.2	55.6	68.25	77.8	123.5
McII	31	25.1	46.2	30.5	28.89	30.4	59.3
Fem	58	51.4	63.4	53.4	52.58	61.6	73.2
Py/Fem	0.45	-	-	0.57	0.45	?	0.19
Hum/Fem	1.29	1.12	1.40	1.15	1.27	1.18	1.66
Uln/Fem	1.22	1.00	1.42	1.04	1.30	1.26	1.69

Supplementary Table 1 Measurements of lengths of selected elements of *Fukuipteryx prima* gen. et sp. nov. in millimeters and comparisons to other non-ornithothoracine taxa. **Abbreviations**: Fem, femur; Hum, humerus; Uln, ulna; McII, metacarpal II; Py, pygostyle.

Supplementary Notes

Geological setting. FPDM-V-9769 was collected in the Kitadani Dinosaur Quarry along the bank of Sugiyama River, Katsuyama, Fukui, Japan (Supplementary Fig. 1). In the quarry, the Kitadani Formation of the Akaiwa Subgroup, the Tetori Group extensively crops out. Here, we briefly summarize geological setting of the formation.

The Tetori Group, exposed in the central part of Honshu (the main island of Japan), consists of various sedimentary deposits representing shallow marine to terrestrial fluvio-lacustrine environments in the Middle Jurassic to the Early Cretaceous[7]. It is generally accepted that deposition of the Tetori Group occurred along the eastern margin of Asia in the Tetori Basin bounded by an orogenic belt to the east and by another mountain range to the west, which separates the Jehol Basin of northeastern China from the Tetori Basin[7].

The Kitadani Formation consists of the uppermost part of the Tetori Group and is generally represented by alternating coarse to fine sandstone and siltstone, and has produced various plant, invertebrate, vertebrate, and trace fossils, whose thorough review is presented elsewhere[8]. FPDM-V-9769 was collected from greenish fine sandstone of the formation which typically yields occasional small plant debris, invertebrates, calcareous nodules and interpreted as overbank deposit of a meandering fluvial system[9]. During the deposition of the Kitadani Formation, the paleoclimate of the region was under a transitional phase from a relatively cold condition as in the Jehol Group to a humid, but locally dry, warm-temperate condition[10]. The age of the Kitadani Formation is suggested Aptian (120 ma) by the most recent, comprehensive study[7].

Additional notes on the surangular. The surangular of birds is a delicate element and is not frequently preserved in the fossil record. Nonetheless, it has been described in some basal birds. Here, we provide additional notes and comparison on the surangular of *Fukuipteryx prima* with other basal birds to support its identification and highlight the morphology.

One of the most detailed descriptions of the surangular in the basal birds comes from the sixth and seventh specimens of *Archaeopteryx*[11]. It bears a long, slightly convex dorsal platform and caudal transverse descending edge with a thick posterodorsal wall. The latter feature is present in *Fukuipteryx prima*. Additionally, the posterior end of the surangular of *Archaeopteryx* (the sixth specimen) exhibits weakly sigmoid, dorsoventrally oriented contact with the articular (Fig. 7 in ref. 11). This feature is observed in the surangular of *F. prima*. The lingual surface of the surangular contacts with the quadrate cotyle at the caudal end in *Archaeopteryx* (Fig. 12 in ref. 11). In *F. prima,* the lingual surface of the surangular bears a shallow depression at the caudal end, which may represent a surangular-quadrate contact similar to that of *Archaeopteryx*.

In non-pygostylian *Jeholornis curvipes*, the surangular is wide and exhibits a groove in its caudal region[12]. The surangular of *Fukuipteryx prima* also has a groove in the caudal region, while it differs from that of *J. curvipes* in being slender and bearing the possible mandibular fenestra.

In enantiornithine *Rapaxiavis pani*, the surangular exhibits two dorsal convexities, and the caudal of which is interpreted as the lateral mandibular process for articulation with the

quadrate (Fig. 4 in ref. 13). The surangular of *Fukuipteryx prima* similarly exhibits a dorsal convexity in its caudodorsal surface, which may represent the lateral mandibular process as in *R. pani.*

The surangular of *Ichthyornis dispar* has been figured in detail[14] (Extended Data Figs. 5, 7, and 9 in ref. 14). The surangulars of *I. dispar* and *Fukuipteryx prima* commonly exhibit the lateral bar dorsally bounding the mandibular fossa lingually (incomplete in *F. prima*) and the thickened caudal portion. On the other hand, the surangular of *I. dispar* is generally more robust than that of *F. prima*. Particularly, the rostral end of the dorsal process (process rostralis in ref. 14) is apparently slenderer in *F. prima*.

Morphological character descriptions. Descriptions of morphological characters used for character scorings in the phylogenetic analysis are provided below.

1. Premaxillae in adults: unfused (0); fused only rostrally (1); completely fused (2) (ORDERED).
2. Maxillary process of the premaxilla: restricted to its rostral portion (0); subequal or longer than the facial contribution of the maxilla (1).
3. Frontal process of the premaxilla: short (0); relatively long, approaching the rostral border of the antorbital fenestra (1); very long, extending caudally near the level of lacrimals (2) (ORDERED).
4. Premaxillary teeth: present throughout (0); present but rostral tip edentulous (1); present but restricted to rostral portion (2); absent (3).

5. Caudal margin of naris: far rostral than the rostral border of the antorbital fossa (0); nearly reaching or overlapping the rostral border of the antorbital fossa (1).

6. Naris longitudinal axis: considerably shorter than the long axis of the antorbital fossa (0); subequal or longer (1).

7. Maxillary teeth: present (0); absent (1).

8. Dorsal (ascending) ramus of the maxilla: present with two fenestrae (the promaxillary and maxillary fenestra) (0); present with one fenestra (1); unfenestrated (2); ramus absent (3). (ORDERED)

9. Caudal margin of choana: located rostrally, not overlapping the region of the orbit (0); displaced caudally, at the same level or overlapping the rostral margin of the orbit (1).

10. Rostral margin of the jugal: away from the caudal margin of the naris (0); or very close to (leveled with) the caudal margin of the naris (1).

11. Contact between palatine and maxilla/premaxilla: palatine contact maxilla only (0); contacts premaxilla and maxilla (1).

12. Vomer and pterygoid articulation: present, well developed (0); reduced, narrow process of pterygoid passes dorsally over palatine to contact vomer (1); absent, pterygoid and vomer do not contact (2).

13. Jugal process of palatine: present (0); absent (1).

14. Contact between palatine and pterygoid: long, craniocaudally overlapping contact (0); short, primarily dorsoventral contact (1).

15. Contact between vomer and premaxilla: present (0); absent (1).

16. Ectopterygoid: present (0); absent (1).

17. Postorbital: present (0); absent (1).

18. Contact between postorbital and jugal: present (0); absent (1).

19. Quadratojugal: sutured to the quadrate (0); joined through a ligamentary articulation (1).

20. Lateral, round cotyla on the mandibular process of the quadrate (quadratojugal articulation): absent (0); present (1).

21. Contact between the quadratojugal and squamosal: present (0); absent (1).

22. Squamosal incorporated into the braincase, forming a zygomatic process: absent (0); present (1).

23. Squamosal, ventral or "zygomatic" process: variably elongate, dorsally enclosing otic process of the quadrate and extending cranioventrally along shaft of this bone, dorsal head of quadrate not visible in lateral view (0); short, head of quadrate exposed in lateral view (1).

24. Frontal/parietal suture in adults: open (0); fused (1).

25. Quadrate orbital process (pterygoid ramus): broad (0); sharp and pointed (1).

26. Quadrate pneumaticity: absent (0); present (1).

27. Quadrate: articulating only with the squamosal (0); articulating with both prootic and squamosal (1).

28. Otic articulation of the quadrate: articulates with a single facet (squamosal) (0); articulates with two distinct facets (prootic and squamosal) (1); articulates with two distinct facets and quadrate differentiated into two heads (2) (ORDERED).

29. Quadrate distal end: with two transversely aligned condyles (0); with a triangular, condylar pattern, usually composed of three distinct condyles (1).

30. Basipterygoid processes: long (0); short (articulation with pterygoid subequal to, or longer than, amount projected from the basisphenoid rostrum) (1).

31. Pterygoid, articular surface for basipterygoid process: concave "socket", or short groove enclosed by dorsal and ventral flanges (0); flat to convex (1); flat to convex facet, stalked, variably projected (2) (ORDERED).

32. Eustachian tubes: paired, lateral, and well-separated from each other (0); paired, close to each other and to cranial midline or forming a single cranial opening (1).

33. Osseous interorbital septum (mesethmoid): absent (0); present (1).

34. Dentary teeth: present (0); absent (1).

35. Dentary tooth implantation: teeth in individual sockets (0); teeth in a communal groove (1).

36. Symphysial portion of dentaries: unfused (0); fused (1).

37. Deeply notched rostral end of the mandibular symphysis: absent (0); present (1).

38. Mandibular symphysis, symphyseal foramina: absent (0); single (1); paired (2).

39. Mandibular symphysis, symphyseal foramen/foramina: opening on caudal edge of symphysis (0); opening on dorsal surface of symphysis (1).

40. Small ossification present at the rostral tip of the mandibular symphysis (intersymphysial ossification): absent (0); present (1).

41. Caudal margin of dentary strongly forked: unforked, or with a weakly developed dorsal ramus (0); strongly forked with the dorsal and ventral rami approximately equal in caudal extent (1).

42. Mandibular ramus sigmoidal such that the rostral tip is dorsally convex and the caudal end is dorsally concave: absent (0); present (1).

43. Cranial extent of splenial: stops well caudal to mandibular symphysis (0); extending to mandibular symphysis, though noncontacting (1); extending to proximal tip of mandible, contacting on midline (2) (ORDERED).

44. Meckel's groove (medial side of mandible): not completely covered by splenial, deep and conspicuous medially (0); covered by splenial, not exposed medially (1).

45. Rostral mandibular fenestra: absent (0); present (1).

46. Caudal mandibular fenestra: present (0); absent (1).

47. Articular pneumaticity: absent (0); present (1).

48. Teeth: serrated crowns (0); unserrated crowns (1).

49. Atlantal hemiarches in adults: unfused (0); fused, forming a single arch (1).

50. One or more pneumatic foramina piercing the centra of mid-cranial cervicals, caudal to the level of the parapophysis-diapophysis: present (0); absent (1).

51. Cervical vertebrae: variably dorsoventrally compressed, amphicoelous (“biconcave”: flat to concave articular surfaces) (0); cranial surface heterocoelous (i.e.,

mediolaterally concave, dorsoventrally convex), caudal surface flat or slightly concave (1); heterocoelous cranial (i.e., mediolaterally concave, dorsoventrally convex) and caudal (i.e., mediolaterally convex, dorsoventrally concave) surfaces (2) (ORDERED).

52. Prominent carotid processes in the intermediate cervicals: absent (0); present (1).
53. Postaxial cervical epipophyses: prominent, projecting further back from the postzygapophysis (0); weak, not projecting further back from the postzygapophysis, or absent (1).
54. Keel-like ventral surface of cervical centra: absent (0); present (1).
55. Prominent (50% or more the height of the centrum's cranial articular surface) ventral processes of the cervicothoracic vertebrae: absent (0); present (1).
56. Thoracic vertebral count: 13-14 (0); 11-12 (1); fewer than 11 (2) (ORDERED).
57. Thoracic vertebrae: at least part of series with subround, central articular surfaces (e.g., amphicoelous/opisthocoelous) that lack the dorsoventral compression seen in heterocoelous vertebrae (0); series completely heterocoelous (1).
58. Caudal thoracic vertebrae, centra, length and midpoint width: approximately equal in length and midpoint width (0); length markedly greater than midpoint width (1).
59. Wide vertebral foramen in the mid-caudal thoracic vertebrae, vertebral foramen/articular cranial surface ratio (vertical diameter) larger than 0.40: absent (0); present (1).

60. Hyposphene-hypantrum accessory intervertebral articulations in the thoracic vertebrae: present (0); absent (1).

61. Lateral side of the thoracic centra: weakly or not excavated (0); deeply excavated by a groove (1); excavated by a broad fossa (2).

62. Cranial thoracic vertebrae, parapophyses: located in the cranial part of the centra of the thoracic vertebrae (0); located in the central part of the centra of the thoracic vertebrae (1).

63. Notarium: absent (0); present (1).

64. Sacral vertebrae, number ankylosed (synsacrum): less than 7 (0); 7 (1); 8 (2); 9 (3); 10 (4); 11 or more (5); 15 or more (6) (ORDERED).

65. Synsacrum, procoelous articulation with last thoracic centrum (deeply concave facet of synsacrum receives convex articulation of last thoracic centrum): absent (0); present (1).

66. Cranial vertebral articulation of first sacral vertebra: approximately equal in height and width (0); wider than high (1).

67. Series of short sacral vertebrae with dorsally directed parapophyses just cranial to the acetabulum: absent (0); present, three such vertebrae (1); present, four such vertebrae (2) (ORDERED).

68. Convex caudal articular surface of the synsacrum: absent (0); present (1).

69. Degree of fusion of distal caudal vertebrae: fusion absent (0); few vertebrae partially ankylosed (intervening elements are well-discernable) (1); vertebrae completely fused into a pygostyle (2) (ORDERED).

70. Free caudal vertebral count: more than 35 (0); 35-26 (1); 25 - 20 (2); 19-9 (3); 8 or less (4) (ORDERED).

71. Procoelous caudals: absent (0); present (1).

72. Distal caudal vertebra prezygapophyses: elongate, exceeding the length of the centrum by more than 25% (0); shorter (1); absent (2) (ORDERED).

73. Free caudals, length of transverse processes: approximately equal to, or greater than, centrum width (0); significantly shorter than centrum width (1).

74. Proximal haemal arches: elongate, at least 3 times longer than wider (0); shorter (1); absent (2) (ORDERED).

75. Pygostyle: longer than or equal to the combined length of the free caudals (0); shorter (1).

76. Cranial end of pygostyle dorsally forked: absent (0); present (1).

77. Cranial end of pygostyle with a pair of laminar, ventrally projected processes: absent (0); present (1).

78. Distal constriction of pygostyle: absent (0); present (1).

79. Ossified uncinate processes in adults: absent (0); present and free (1); present and fused (2).

80. Uncinate process, orientation: perpendicular to rib (0); angled dorsally defining an acute angle with the rib (1).

81. Gastralia: present (0); absent (1).

82. Coracoid shape: rectangular to trapezoidal in profile (0); strut-like (1).

83. Coracoid and scapula articulation: Scapula and coracoid articulation: (0) pit-shaped scapular cotyla developed on the coracoid, and coracoidal tubercle developed on the scapula ("ball and socket" articulation); (1) scapular articular surface of coracoid convex; (2) flat.

84. Scapula: articulated at the shoulder (proximal) end of the coracoid (0); well below it (1).

85. Coracoid, humeral articular (glenoid) facet: dorsal to acrocoracoid process/"biceps tubercle" (0); ventral to acrocoracoid process (1).

86. Humeral articular facets of the coracoid and the scapula: placed in the same plane (0); forming a sharp angle (1).

87. Coracoid, acrocoracoid: straight (0); hooked medially (1).

88. Laterally compressed shoulder end of coracoid, with nearly aligned acrocoracoid process, humeral articular surface, and scapular facet, in dorsal view: absent (0); present (1).

89. Procoracoid process on coracoid: absent (0); present (1).

90. Lateral margin of coracoid: concave (0); nearly concave to straight for most part and the convex portion is restricted at sternal end, which measures less than half the

width of sternal end (1); strongly convex, and the convex portion measuring more than half the sternal end (2).

91. Broad, deep fossa on the dorsal surface of the coracoid (dorsal coracoidal fossa): absent (0); present (1).

92. Supracoracoidal nerve foramen of coracoid: centrally located (0); displaced toward (often as an incisure) the medial margin of the coracoid (1); displaced so that the nerve no longer passes through the coracoid (absent) (2) (ORDERED).

93. Coracoid, medial surface, strongly depressed elongate furrow at the level of the passage of n. supracoracoideus: absent (0); present (1).

94. Supracoracoid nerve foramen, location relative to dorsal coracoidal fossa: above fossa (0); inside fossa (1).

95. Coracoid, sternolateral corner: unexpanded (0); expanded (1); well developed squared-off lateral process (sternocoracoidal process) (2); present and with a distinct omal projection (hooked) (3).

96. Scapular shaft: straight, both dorsal and ventral margins straight (0); straight shaft with convex dorsal margin and straight ventral margin (1); the scapular shaft sagittally curved (2).

97. Scapula, length: shorter than humerus (0); as long as or longer than humerus (1).

98. Scapular acromion process: in lateral or costal view, strongly projecting craniodorsally, forming a large angle with the proximal shaft of the scapular (0); nearly parallel to the shaft of the scapular (1).

99. Scapula, acromion process: projected cranially surpassing the articular surface for coracoid (0); projected less cranially than the articular surface for coracoid (1).

100. Scapula, acromion process, in costal or lateral aspect: straight and tapered toward cranial end (0); barely tapered with a blunt end (1); laterally hooked tip (2).

101. Proximal end of scapula, pit between acromion and humeral articular facet (scapular fossa): absent (0); present (1).

102. Costal surface of scapular blade with prominent longitudinal furrow: absent (0); present (1).

103. Scapular caudal end: blunt (may or may not be expanded) (0); sharply tapered (1).

104. Furcular, shape: boomerang-shaped (0); V to Y-shaped (1); U-shaped (2).

105. Furcula interclavicular angle: approximately 90° (0); less than 70° (1). The interclavicular angle is measured as the angle formed between three points, one at the omal end of each rami and the apex located at the clavicular symphysis.

106. Dorsal and ventral margins of the furcula: subequal in width (0); ventral margin distinctly wider than the dorsal margin so that the furcular ramus appears concave laterally (1).

107. Hypocleideum: absent (0); present as a tubercle or short process (1); present as an elongate process approximately 30% rami length (2); hypertrophied, exceeding 50% rami length (3) (ORDERED).

108. Sternum: unossified (0); partially ossified, coracoidal facets cartilaginous (1); fully ossified (2).

109. Ossified sternum: two flat plates (0); single flat element (1); single element, with slightly raised midline ridge (2); single element, with projected carina (3).

110. Sternal carina: near to, or projecting rostrally from, the cranial border of the sternum (0); not reaching the cranial border of the sternum (1).

111. Sternum, caudal margin, number of paired caudal trabecula: none (0); one (1); two (2).

112. Sternum, outermost trabecula, shape: tips terminate cranial to caudal end of sternum (0); tips terminate at or approaching caudal end of sternum (1); tips extend caudally past the termination of the sternal midline (2).

113. Prominent distal expansion in the outermost trabecula of the sternum: absent (0); present, simple bulb-like (1); fan-shaped expansion (2); triangular expansion with an acute medial angle (3); branched (4).

114. Rostral margin of the sternum broad and rounded: absent (0); present (1).

115. Sternum, coracoidal sulci spacing on cranial edge: widely separated mediolaterally (0); adjacent (1); crossed on midline (2).

116. Costal facets of the sternum: absent (0); present (1).

117. Sternal costal processes: three (0); four (1); five (2); six (3); seven (4); eight (5) (ORDERED).

118. Sternal midline, caudal end: blunt W-shape (0); V-shape (1); elongate straight projection (xiphoid process) (2); xiphoid process slightly flared mediolaterally

(3); xiphoid process distal end strongly flared with prominent medial and lateral projections (4); rounded (5).

119. Sternum, caudal half, paired enclosed fenestra: absent (0); present (1).

120. Sternum, dorsal surface, pneumatic foramen (or foramina): absent (0); present (1).

121. Proximal and distal humeral ends: twisted (0); expanded nearly in the same plane (1).

122. Humeral head: concave cranially and convex caudally (0); globe shaped, craniocaudally convex (1).

123. Proximal margin of the humeral head concave in its central portion, rising ventrally and dorsally: absent (0); present (1).

124. Humerus, proximocranial surface, well-developed circular fossa on midline: absent (0); present (1).

125. Humerus with distinct transverse ligamental groove: absent (0); present (1).

126. Humerus, ventral tubercle projected caudally, separated from humeral head by deep capital incision: absent (0); present (1).

127. Pneumatic fossa in the caudoventral corner of the proximal end of the humerus: absent or rudimentary (0); well developed (1).

128. Humerus, deltopectoral crest: projected dorsally (the plane of the crest is coplanar to the cranial surface of the humerus) (0); projected cranially (1).

129. Humerus, deltopectoral crest: less than shaft width (0); approximately same width (1); prominent and subquadrangular (i.e., subequal length and width) (2).

130. Humerus, deltopectoral crest, perforated by a large fenestra: absent (0); present (1).

131. Humerus, bicipital crest: little or no cranial projection (0); developed as a cranial projection relative to shaft surface in ventral view (1); hypertrophied, rounded tumescence (2).

132. Humerus, distal end of bicipital crest, pit-shaped fossa for muscular attachment: absent (0); craniodistal on bicipital crest (1); directly ventrodistal at tip of bicipital crest (2); caudodistal, variably developed as a fossa (3).

133. Distal end of the humerus very compressed craniocaudally: absent (0); present (1).

134. Humerus, demarcation of muscle origins (e.g., m. extensor metacarpi radialis in Aves) on the dorsal edge of the distal humerus: no indication (0); a pit or a tubercle (1); a variably projected scar-bearing tubercle (dorsal supracondylar process) (2).

135. Well-developed brachial depression on the cranial face of the distal end of the humerus: absent (0); present (1).

136. Well-developed olecranon fossa on the caudal face of the distal end of the humerus: absent (0); present (1).

137. Humerus, distal end, caudal surface, groove for passage of m. scapulotriceps: absent (0); present (1).

138. Humerus, m. humerotricipitalis groove: absent (0); present as a well-developed ventral depression contiguous with the olecranon fossa (1).

139. Humerus, distal margin: approximately perpendicular to long axis of humeral shaft (0); ventrodistal margin projected significantly distal to dorsodistal margin, distal margin angling strongly ventrally (sometimes described as a well-projected flexor process) (1).

140. Humeral distal condyles: mainly located on distal aspect (0); on cranial aspect (1).

141. Humerus, long axis of dorsal condyle: at low angle to humeral axis, proximodistally oriented (0); at high angle to humeral axis, almost transversely oriented (1).

142. Humerus, distal condyles: subround, bulbous (0); weakly defined, “straplike” (1).

143. Humerus, ventral condyle: length of long axis of condyle less than the same measure of the dorsal condyle (0); same or greater (1).

144. Ulna: shorter than humerus (0); nearly equivalent to or longer than humerus (1).

145. Ulnar shaft, radial-shaft/ulnar-shaft ratio: larger than 0.70 (0); smaller than 0.70 (1).

146. Ulna, cotylae: dorsoventrally adjacent (0); widely separated by a deep groove (1).

147. Ulna, dorsal cotyla strongly convex: absent (0); present (1).

148. Ulna, bicipital scar: absent (0); developed as a slightly raised scar (1); developed as a conspicuous tubercle (2).

149. Proximal end of the ulna with a well-defined area for the insertion of m. brachialis anticus: absent (0); present (1).

150. Semilunate ridge on the dorsal condyle of the ulna: absent (0); present (1).

151. Shaft of radius with a long longitudinal groove on its ventrocaudal surface: absent (0); present (1).

152. Ulnare: heart-shaped with little differentiation into short rami (0); U-shaped to V-shaped, well-developed rami (1).

153. Ulnare, ventral ramus (crus longus, Baumel and Witmer, 1993): shorter than dorsal ramus (crus brevis) (0); same length as dorsal ramus (1); longer than dorsal ramus (2).

154. Semilunate carpal and proximal ends of metacarpals in adults: unfused (0); semilunate fused to the alular (I) metacarpal (1); semilunate fused to the major (II) and minor (III) metacarpals (2); fusion of semilunate and all metacarpals (3). Any specimen that is inferred to be a juvenile should be scored as a "?" in order to account for the possibility of ontogenetic change.

155. Semilunate carpal, position relative to the alular metacarpal (I): over entire proximal surface (0); over less than one-half proximal surface or no contact present (1).

156. Carpometacarpus, proximal ventral surface: flat (0); raised ventral projection contiguous with minor metacarpal (1); pisiform process forming a distinct peg-like projection (2).

157. Carpometacarpus, ventral surface, supratrochlear fossa deeply excavating proximal surface of pisiform process: absent (0); present (1).

158. Round-shaped alular metacarpal (I): absent (0); present (1).

159. Alular metacarpal (I), extensor process: absent, no cranioproximally projected muscular process (0); present, tip of extensor process just surpassed the distal articular facet for phalanx 1 in cranial extent (1); tip of extensor process

conspicuously surpasses articular facet by approximately half the width of facet, producing a pronounced knob (2); tip of extensor process conspicuously surpasses articular facet by approximately the width of facet, producing a pronounced knob (3) (ORDERED).

160. Alular metacarpal (I), distal articulation with phalanx I: ginglymoid (0); shelf (1); ball-like (2).

161. Metacarpal III, craniocaudal diameter as a percentage of same dimension of metacarpal II: approximately equal or greater than 50% (0); less than 50% (1).

162. Proximal extension of metacarpal III: level with metacarpal II (0); ending distal to proximal surface of metacarpal II (1).

163. Intermetacarpal process or tubercle on metacarpal II: absent (0); present as scar (1); present as tubercle or flange (2).

164. Intermetacarpal space: absent or very narrow (0); at least as wide as the maximum width of minor metacarpal (III) shaft (1).

165. Intermetacarpal space: reaches proximally as far as the distal end of metacarpal I (0); terminates distal to end of metacarpal I (1).

166. Distal end of metacarpals: unfused (0); partially or completely fused (1).

167. Minor metacarpal (III) projecting distally more than the major metacarpal (II): absent (0); present (1).

168. Alular digit (I), phalanx 1, distal extension relative to the major metacarpal (II): beyond the distal end of major metacarpal (0); approximately equal in distal

extension (1); shorter than the distal end but beyond half of the major metacarpal (2); terminating less than half of the major metacarpal (3) (ORDERED).

169. Proximal phalanx of major digit (II): of normal shape (0); flat and craniocaudally expanded (1).

170. Major digit (II), phalanx 1, “internal index process” (Stegmann, 1978) on caudodistal edge: absent (0); present (1).

171. Second phalanx of major digit (II): longer than proximal phalanx (0); shorter than or equivalent to proximal phalanx (1).

172. Ungual phalanx of major digit (II): present (0); absent (1).

173. Ungual phalanx of major digit (II): larger or subequal to other manual unguals (0); smaller than the alular ungual but larger than that of the minor (III) digit, and the ungual of the minor digit may or may not present (1); smaller than the unguals of the alular and minor digits (2).

174. Proximal phalanx of the minor digit (III) much shorter than the remaining non-ungual phalanges of this digit: absent (0); present (1).

175. Ungual phalanx of minor digit (III): present (0); absent (1).

176. Length of manus (semilunate carpal + major metacarpal and digit) relative to humerus: longer (0); subequal (1); shorter (2) (ORDERED).

177. Intermembral index = (length of humerus + ulna)/(length of femur + tibiotarsus): less than 0.7, flightless (0); between 0.7 and 0.9 (1); between 0.9 and 1.1 (2); greater than 1.1 (3).

178. Pelvic elements in adults, at the level of the acetabulum: unfused or partial fusion (0); completely fused (1).

179. Ilium/ischium, distal co-ossification to completely enclose the ilioischiadic fenestra: absent (0); present (1).

180. Preacetabular process of ilium twice as long as postacetabular process: absent (0); present (1).

181. Preacetabular ilium: approach on midline, open, or cartilaginous connection (0); co-ossified, dorsal closure of "iliosynsacral canals" (1).

182. Ilium, m. cuppedicus fossa as broad, mediolaterally oriented surface directly cranioventral to acetabulum: present (0); surface absent, insertion variably marked by a small entirely lateral fossa cranial to acetabulum (1).

183. Preacetabular pectineal process (Baumel and Witmer, 1993): absent (0); present as a small flange (1); present as a well-projected flange (2) (ORDERED).

184. Small acetabulum, acetabulum/ilium length ratio equal to or smaller than 0.11: absent (0); present (1).

185. Prominent antitrochanter: caudally directed (0); caudodorsally directed (1).

186. Postacetabular process shallow, less than 50% of the depth of the preacetabular wing at the acetabulum: absent (0); present (1).

187. Iliac brevis fossa: present (0); absent (1).

188. Ischium: two-thirds or less the length of the pubis (0); more than two-thirds the length of the pubis (1).

189. Obturator process of ischium: prominent (0); reduced or absent (1).

190. Ischium, caudal demarcation of the obturator foramen: absent (0); present, developed as a small flange or raised scar contacting/fused with pubis and demarcating the obturator foramen distally (1).

191. Ischium with a proximodorsal (or proximocaudal) process: absent (0); present (1).

192. Ischiadic terminal processes forming a symphysis: present (0); absent (1).

193. Orientation of proximal portion of pubis: cranially to subvertically oriented (0); retroverted, separated from the main synsacral axis by an angle ranging between 65º and 45º (1); more or less parallel to the ilium and ischium (2) (ORDERED).

194. Pubic pedicel: cranioventrally projected (0); ventrally or caudoventrally projected (1).

195. Pubic pedicel of ilium very compressed laterally and hook-like: absent (0), present (1).

196. Pubic shaft laterally compressed throughout its length: absent (0); present (1).

197. Pubic apron: present (0); absent (absence of symphysis) (1).

198. Pubic foot: flaring into simple round shape (0); triangular shape with a pointed caudal tip and caudoventally directed with respect to the distal pubic shaft (1); the caudal tip recurved caudodorsally with respect to the distal pubic shaft (2); absent (3).

199. Femur with distinct fossa for the capital ligament: absent (0); present (1).

200. Femoral neck: present (0); absent (1).

201. Femoral anterior trochanter: separated from the greater trochanter (0); fused to it, forming a trochanteric crest with a laterally curved edge (1); fused to it, forming a trochanteric crest with a flattened edge (2).

202. Femoral trochanteric crest: projects proximally beyond femoral head (0); equal in proximal projection (1); does not project beyond femoral head (2).

203. Femoral posterior trochanter: present, developed as a slightly projected tubercle or flange (0); hypertrophied, “shelf-like” conformation (1); absent (2).

204. Femur with prominent patellar groove: absent (0); present as a continuous extension onto the distal shaft (1); present and separated from the shaft by a slight ridge, giving it a pocketed appearance (2).

205. Femur: ectocondylar tubercle and lateral condyle separated by deep notch (0); ectocondylar tubercle and lateral condyle contiguous but without developing a tibiofibular crest (1); tibiofibular crest present, defining laterally a fibular trochlea (2) (ORDERED).

206. Caudal projection of the lateral border of the distal end of the femur, proximal and contiguous to the ectocondylar tubercle/tibiofibular crest: absent (0); present (1).

207. Femoral popliteal fossa distally bounded by a complete transverse ridge: absent (0); present (1).

208. Fossa for the femoral origin of m. tibialis cranialis: absent (0); present (1).

209. Tibia, calcaneum, and astragalus: unfused or poorly co-ossified (sutures still visible) (0); complete fusion of tibia, calcaneum, and astragalus (1).

210. Round proximal articular surface of tibiotarsus: absent (0); present (1).

211. Tibiotarsus, proximal articular surface: flat (0); angled so that the medial margin is elevated with respect to the lateral margin (1).

212. Tibiotarsus, cnemial crests: absent (0); present, one (1); present, two (2).

213. Tibia, caudal extension of articular surface for distal tarsals/tarsometatarsus: absent, articular restricted to distalmost edge of caudal surface (0); well-developed caudal extension, sulcus cartilaginis tibialis of Aves (Baumel and Witmer, 1993), distinct surface extending up the caudal surface of the tibiotarsus (1); with well-developed, caudally projecting medial and lateral crests (2) (ORDERED).

214. Extensor canal on tibiotarsus: absent (0); present as an emarginate groove (1); groove bridged by an ossified supratendinal bridge (2) (ORDERED).

215. Tibia/tarsal-formed condyles: medial condyle projecting farther cranially than lateral condyle (0); equal in cranial projection (1).

216. Tibia/tarsal-formed condyles, mediolateral widths: medial condyle wider (0); approximately equal (1); lateral condyle wider (2) (ORDERED).

217. Tibia/tarsal-formed condyles: gradual sloping of condyles towards midline of tibiotarsus (0); no tapering of either condyle (1).

218. Proximal end of the fibula: prominently excavated by a medial fossa (0); nearly flat (1).

219. Fibula, tubercle for m. iliofibularis: craniolaterally directed (0); laterally directed (1); caudolaterally or caudally directed (2) (ORDERED).

220. Fibula, distal end reaching the proximal tarsals: present (0); absent (1).

221. Distal tarsals in adults: free (0); completely fused to the metatarsals (1). Any specimen that is inferred to be a juvenile should be scored as a "?" in order to account for the possibility of ontogenetic change.

222. Metatarsals II-IV, intermetatarsal fusion: absent or minimal co-ossification (0); partial fusion, sutural contacts easily discernible (1); completely or nearly completely fused, sutural contacts absent or poorly demarcated (2) (ORDERED).

223. Proximal end of metatarsus: plane of articular surface perpendicular to longitudinal axis of metatarsus (0); strongly inclined dorsally (1).

224. Metatarsal V: present (0); absent (1).

225. Proximal end of metatarsal III: in the same plane as metatarsals II and IV (0); plantarly displaced with respect to metatarsals II and IV (1).

226. Tarsometatarsal proximal vascular foramen/foramina: absent (0); one between metatarsals III and IV (1); two (2).

227. Metatarsals, relative mediolateral width: metatarsal IV approximately the same width as metatarsals II and III (0); metatarsal IV narrower than metatarsals II and III (1); metatarsal IV greater in width than either metatarsal II or III (2).

228. Well-developed tarsometatarsal intercotylar eminence: absent (0); present, low and rounded (1); present, high and peaked (2).

229. Tarsometatarsus, projected surface and/or grooves on proximocaudal surface (associated with the passage of tendons of the pes flexors in Aves; hypotarsus):

absent (0); developed as caudal projection with flat caudal surface (1); projection, with distinct crests and grooves (2); at least one groove enclosed by bone caudally (3) (ORDERED).

230. Plantar surface of tarsometatarsus excavated: absent (0); present (1).

231. Tarsometatarsal distal vascular foramen completely enclosed by metatarsals III and IV: absent (0); present (1).

232. Metatarsal I: straight (0); J-shaped, the articulation of the hallux is located on the same plane as the attachment surface of the metatarsal I (1); J-shaped; the articulation of the hallux is perpendicular to the attachment surface (2); the distal half of the metatarsal I is laterally deflected so that the laterodistal surface is concave (3).

233. Metatarsal II tubercle (associated with the insertion of the tendon of the m. tibialis cranialis in Aves): absent (0); present, on approximately the center of the proximodorsal surface of metatarsal II (1); present, developed on lateral surface of metatarsal II, at contact with metatarsal III or on lateral edge of metatarsal III (2) (ORDERED).

234. Metatarsal II, distal plantar surface, fossa for metatarsal I (fossa metatarsi I; Baumel and Witmer, 1993): absent (0); shallow notch (1); conspicuous ovoid fossa (2) (ORDERED).

235. Relative position of metatarsal trochleae: trochlea III more distal than trochleae II and IV (0); trochlea III at same level as trochlea IV, both more distal than trochlea

II (1); trochlea III at same level as trochleae II and IV (2); distal extent of trochlea III intermediate to trochlea IV and II where trochlea IV projects furthest distally (3).

236. Metatarsal II, distal extent of metatarsal II relative to metatarsal IV: approximately equal in distal extent (0); metatarsal II shorter than metatarsal IV but reaching distally farther than base of metatarsal IV trochlea (1); metatarsal II shorter than metatarsal IV, reaching distally only as far as base of metatarsal IV trochlea (2).

237. Distal tarsometatarsus, trochlea in distal view: aligned in a single plane (0); metatarsal II slightly displaced plantarly with respect to III and IV (1); metatarsal II strongly displaced plantarly in respect to III and IV, such that there is little or no overlap in medial view (2).

238. Trochlea of metatarsal II broader than the trochlea of metatarsal III: absent (0); present (1).

239. Metatarsal III, trochlea in plantar view, proximal extent of lateral and medial edges of trochlea: trochlear edges approximately equal in proximal extent (0); medial edge extends farther (1).

240. Distal end of metatarsal II strongly curved medially: absent (0); present (1).

241. Digit IV phalanges in distal view, medial trochlear rim enlarged with respect to lateral trochlear rim: absent (0); present (1); greatly enlarged with the lateral trochlea reduced to a rounded peg (2).

242. Completely reversed hallux (arch of ungual phalanx of digit I opposing the arch of the unguals of digits II-IV): absent (0); present (1).

243. Size of claw of hallux relative to other pedal claws: shorter, weaker, and smaller (0); similar in size (1); longer, more robust, and larger (2).

244. Alula: absent (0); present (1).

245. Fan-shaped feathered tail composed of more than two elongate retrices: absent (0); present (1).

246. Sternum, outermost trabecula: mainly parallel to the long axis of the sternum (0); clearly directed laterally (1).

247. Distal end of furcula relative to sternal margin of coracoid: proximal to or level with the sternal margin of the coracoid (0); well beyond the sternal end of the coracoid (1). When coracoid and furcula are not remained in natural position, then their proximodistal lengths are compared.

248. Scapula and coracoid: fused (0); unfused (1).

249. Scapula, acromion process length relative to the length of the humeral articular facet: less than half (0); nearly equivalent (1); longer but less than two times (2); more than two times longer (3) (ORDERED).

250. Alular digit (I), phalanx 1: longer than the phalanx 1 of digit II (0); shorter than or equivalent to the phalanx 1 of digit II (1).

251. Coracoid, width of the sternal end relative to the length along the shaft: approximately half or greater (0); between half to 1/3 (1); less than 1/3 (2).

252. Coracoid, sternal margin: convex (0); nearly straight (1); concave (2);

253. Humerus, deltopectoral crest, distal end recedes abruptly with the humeral shaft: present (0); absent (1)

254. Tibia/tarsal-formed condyles, intercondylar groove: mediolaterally broad, approximately 1/3 with of anterior surface (0); less than 1/3 width of anterior surface (1).

255. Metatarsal IV, distal extension of the metatarsal IV relative to the metatarsal III: shorter and proximal to the proximal margin of the trochleae III (0); shorter but reaching distally further than the proximal margin of the trochleae III (1); approximately equal or surpassing the trochleae III (2)

256. Reduced claw in digit IV: absent (0); present (1).

257. The ratio (tibiotarsus length/tarsometatarsus length): 2 or larger (0); between 2 and 1.6 (1); smaller than 1.6 (2). When distal tarsals are not fused with metatarsals, metatarsal III length is used.

258. Pedal digit, Penultimate phalanx, longer than preceding phalanges in each digit: absent (0); present (1).

259. Proximal phalanx of hallux, the longest non-ungual phalanx: absent (0); present (1).

260. Phalanx in digit IV: not as follows (0), the second and the third phalanges reduced and significantly shorter than the fourth phalanx (1), as before but with the proximal phalanx reduced to be nearly equal in length with the second and third phalanx (2).

261. Ungual in digit III, length relative to the tarsometatarsus: less than 20% the length of tarsometatarsus (0); 20% – 40% (1); extremely elongated and measuring more than 40% the length of tarsometatarsus (2); When metatarsals are not fused with distal tarsals, the length of metatarsal III is used. The length of ungual represents the linear distance between proximal end (position equivalent to flexor process) and tip of the sheath. If the sheath is not preserved or disarticulated with bony ungual, it should be scored as "?" (ORDERED).

262. Surangular: not as follows (0); dorsal margin concave and ventral margin convex (1); sigmoid (2).

263. Premaxilla, preorbital portion occupying 60% or more the skull length: absent (0); present (1).

264. Pedal digit II: not as follows (0); much robust than the other digits (1)

265. Alular digit (I): long, exceeding the distal end of the major metacarpal (0); subequal (1); short, not surpassing this metacarpal (2) (ORDERED).

266. Tarsometatarsus, length compared to femur length: 0.6 or fewer (0); 0.8-0.6 (1); 1-0.8 (2);1 or greater (3).

267. Quadratojugal, shape: without horizontal process posterior to ascending process (reversed "L" shape) (0); with horiziontal posterior process (i.e., inverted 'T' or 'Y' shape) (1).

268. Jugal and quadratojugal, fusion: absent (0); present, the two bones are not distinguishable from one another (1) (ORDERED).

269. Caudal vertebrae, change in morphology of free caudals along the tail: present, with distinct transition point from shorter centra with long transverse processes proximally to longer centra with small or no transverse processes distally (0); absent, vertebrae homogeneous in shape, without transition point (1).

270. Caudal vertebrae, location of transition point along the tail: begins distal to the 10th caudal vertebra (0); between the 7th and 10th caudal vertebra (1); proximal to the 7th caudal vertebra (2) (ORDERED).

271. Coracoid and scapula, angle between bones at glenoid: greater than or equivalent to 90° (0); smaller than 90° (1).

272. Ischium, distal end: continuous with the proximal shaft, rendering the cranial margin of the ischium straight or weakly convex (0); directed cranioventrally toward the pubis, rendering the cranial margin concave (1).

273. Ischium, caudal margin with a dorsal process: located proximal to or close to the midpoint of the caudal margin (0); distal to the midpoint of the caudal margin (1).

274. Sternum, cranial margin with a pair of craniolateral processes: absent (0); present (1).

275. Premaxilla corpus: dorsoventral height greater than or equal to craniocaudal length (0); dorsoventral height smaller than craniocaudal length (1).

276. Furcula, omal tip: blunt or expanded (0); tapered (1).

277. Alular metacarpal, cranial margin: expanded cranioproximally and craniodistally, and constricted just before flare of articulation with digit (0); broadly convex (1).

278. Alular digit, phalanx 1: straight (0); bowed (1).

279. Major digit, phalanx 2: straight (0); bowed (1).

280. Minor digit: with four phalanges (0); less than four phalanges (1).

Character scorings. Character scorings for the taxa used in the phylogenetic analysis are provided below. The character scorings for previously-described taxa follow those presented in ref. 2. and we collected those for *Fukuipteryx prima* in this study.

Dromaeosauridae

000000000000000000000000000000000000??0000000000000000000000000000000
01????1?00?0000000000?000000000000020?[01]??0010000000000001000000000000000
100000000??000?000000000000000[01]0000000[01]00000000000010000[01]00?00000
00001000000000000000000[01]000000000000?0000000000[01]00000100010010000
000000

Archaeopteryx

00000000000000?0010010?00?000??0?000???0000000?1000?100000??00000?0002000
1????0?00?0000000000?0000?0?0000000????????????000?000110000000000???0000
0000??0010000010000000000000002000000001?0001?010002?10?00000?0001?0??0??0
0000000000000?0?00?000100?0000000?001000100001000200?100000

Jeholornis

[01]?23111?????????????0?????????????00?????000?00?1??0????00??10000????010000
??????01001?000001??1201?0??1??00[12]?????????????0000000000?0[01]00??010111
10?00000121100100001000200001001300000000011?101?010001????0?1?0?0??0[01]0?
10??1100000000??00?0200?0010?00?1?0010010100?000000002100000100

Chongmingia

??00????????[01]???????00????

???0??0????000???000??????0000?????????????001??00010??00?0??01?0?10????000?2

1??00000010001001????[01]30??????????????[12]??000?111?0????1?001??00??110000

0000002??0100?001????00?1?[01]111?10001??001????0????0010?

Sapeornis

1010010[01]?0??????0010?1?0?????????1?0????00??0??1????100[01]01??10010?00240

00000?00?00?011000000??020?0???10002??????????????00??0001100000???0100111?

???00??21100001000?002001010103000 0??0?110101111?001010??0100?1000?0?111?1

1000000000000?0000?0011???00?00000001010100000001?000?000101

Confuciusornis_sanctus

10231112?0?????00010110000120???11?112101001100?101?101[01]011120010000240

00?00001?01?0?0?0000?0?020?0?00000002[12]10??0112100000000000210?0000010100

0010021000121 0?00011000000000002100200000000?10101111000310112020 1?1?0010

000?1110000100010210020000001000?00?000001000011000000001?0000101010

Changchengornis_hengdaoziensis

1?23111??0????????1??????0???????1?11??0?0??100???[12]?????????2??1???024??0?0

000??01?0?0?000????020?0??0?000021?0??01??10?0?0????02??????????????01?????0

????0?0000?0??000000021002???0???????????[12]???03???????????0?????????110000

?0?0?02??0100?0011?0?00?0?00?001001100001??1?00???01000

Eoconfuciusornis_zhengi

[12]?231?12?0??????0????????????????1?01??000??100????0?0?00???0??1???0241???

0??0??00?0???000000?020????0000?02[12]???????????0?00000?100?0???000????01??

???0??01??00001?0?0000000210020??0??0?????????1000[12]??????????0??????10????

0?000000002?00200?001000???0??0001010010000001??1?????101010

Confuciusornis_dui

??23111????????????????0??????????1???????0??1?????????????????[012]????14?10?0

??0??0??????????????????????????2[12]?0??0???10?0?0??00021?????????????01?????0

???1??0[01]00?00?000??0021002????????????????????????[02]?????????1???????10??

??0?00????0[12]00?0010?0????0??0?1000011?00[01]0001?0???1??010

Boluochia

?1[12][02]???2??????

??1?????????????????????????????[12][12]?[12]??????20????????????????????????????

???110101???02??????????0???????????0[01]?1

00100002??3200?0011??1????????2?20??????????1?????1?????

Concornis

??01??11????????0?01??????

?111110?02?11?00?1?????1112221222111?20?00111??0[01]0211??????1????1112??????

??????0??0???30010[01]0122??????????11?1?[12]??00?0?????????1111???????1110?00

10??0[01]??0000?001???01131110?0?2??1???022??1?1??1?0??01

Elsornis

???[01]??10?0?1?10???????????????

????11111000212 1?000001010111[23]22010?11??[12]0?00110000000[12]0[12]0000111
??0100[12]1?1??31?????0?[01]?01???
?????????????????????????????1111?121?????????????????1??0?0????

Eoalulavis

???00111200??2?0???????????????0
??1111101021110000001 0?111?312?0?0010?40?0011110010211001001111111 1?21?1??
???????0??1?01?001????2???0?????1??????????????1?1????????????????????????????
???????????1??1121111???????????1?????1??0?0?101

Cathayornis

[12]0[12]0??0??????????????????????????000????00?????1??1??1??0???11?2????240?0
?0111???1111?0??20???000001?0111?322122311??20?0011?1?010211[01]01011111011
????110?31100010?[01]000130010101220000 00??11??????100???12????1????[01]???
??1?1??????????????????????????0?1111111????????0?2???1?10?01?1101

Eocathayornis

???0???2???????????????0???0?????000????00?????1????????00????????????????????
??11?????02????000000??0????22121311??20??01??1?000???[01]????11???10??????0?
31??0010??000120010001 1??
??????????????0?111111????????????2?????1??01?1101

Eoenantiornis

[01]010??02????????????????0??????????00?????????????1???????????????[012]????2402
0?????1?0111110101?11000?001??11122[123]?2??110?2000?1??1?000?????????????1

1?????10?31??0000?00001200101012 2???0???????????[12]??001????1???????????????

??11??0?10??????0??1?0?1110011[23]1011?[01]?1?0??20?11??1?10?0001101

Gobipteryx

10[12]3101200021?10????????0???0?1??1?[01]0???10???10??????1?????????[012]10??

[12]401?????????1111?010?1???0??0010??111[23]?????????????0??????0??????0??????

??11???111?????????0??0?01?00??????20??????????????10?03???????????10[01]10?0

11?110010?100??11?0??1?1??????????2??1????????0???????1???1?????

Longipteryx

?122111??????????????????????????000????00??01?1??1011?????????2????24??0?01?1

1?01?1110100????000?000?111132[23]?21111??20??0110??0002??[01]?0?011?1?11???

???0?01100000100?01200100011300000????1111011[12]11001?0?[12]1??0????0????0?1

?1?0?1000000001?32000001???1?1211?1?2?2????01?11??1?1000101101

Longirostravis

[01]022??1??????????????????????????00?????00??01?1??1?????0?????01????24??0?0

1110?0111????00?2??000000??0111[123]2[123]?224110?20??01??1??00???[01]???????

??10??????0?31??10?0?[01]01012001??0122??0?????1??10?1?11????0?1??????1?00???

??1?11[01]?10010??0?2?0100?00????0?1??111?1?1????01??1??1?1??010????

Neuquenornis

??0???11??????????????????

?1?1????021111000????1?1111230[12]?1?1?????0????11?????1?01?01????11?1??11??3

1??10?0?[01]0001??????????????????????????????????1110???????1????????10??0?1??1

??2?00?011001[12]????1??2[12]0???1?0????0??????1????01???

Pengornis

00[01]01101?0??????01?????0?1???????000???0?0?????11?2??1??0?1?10?1????240?0[12]??????01?11???00?????0??02???11?2?????????????001?0100101??[01]?100?1???11??????1[02][23]111???0?01001?0010?0?[12]3??????????????????02?01???????1??[01]?01[12]11?1100100100101200201?00?????011?100?[01]011?02101?0??1?1???10??01

Eopengornis

0000110[012]?0?????????????0?????????00000?000?????1??10?0??00?????????24??0?1?????01211101000???000?02?00111321?110?1??10?0010010000??10?0001???10??????0??1[01]?00?0?01001200101010 3?????????????????000???????0????100??????0???0001????1??0101?0012?000111101?1011102?0101??1?1??0101101

Protopteryx

[12]?[12][02]1??[012]?????????????????????????00??????00????1???????10?????01????14????0??1???1111?0?11?[12]??200?00??0111322111011?[12]10???1????010?????????????11?????????1??000??[01]000110000001 0???00???11?????????0[12]????1?????0???????????00??0010??00??0[01]01?001110011?0100?[01]101101?0000??1?1000101101

Rapaxavis

[01]022??1[012]?????????????????????????000???0?0???1?1?????1??01??11?1???024??0?0?110?01????0?00?[12]??0??001???11?322122411??20??0100??0000??[01]0???1111?10???1??0??11?00?010000130011?01230000?0??111101?111?0100????????0?0[01]?0?11??100?10000??002?0100?0011??0?121011?10110210 1021??1?100?101101

Shanweiniao

[01]?[12][23]??1????????????????????????????0????00??01??????1???????????????24?

????1?1??01?11???000[12]??000000??011?22[123]?211?[01]??20???1?????00????????1

1???11????1?????????????????20?11???22?????????1??????1????0??????0??1?????????

?110??0000????1?[01][01]?0?0?1[01]?10?12?111?1011?21010[01]???1?1??010??01

Vescornis

?0?????2????????????????????????0000???00[12]001?1??21?1??0???1??2????2??????

?00??01?1??0?02?[12]??000001??011?322120111??20?00110??0101?1[01]0???1111111

??????1[02]011010?00[01]0?0130010[01]01220??0??????????????????????????0??[01]

????????00??0010??011?[01]201?1011??11121[01]11?[01]1110?00?021??1?1??0?01101

Qiliania

??

??

???????????????10????00?10101?2?0003?0??11200?00?110000?11100?001001?11?[01]

?20?0?1[01]??????????1?120021??1?2?????00???????

Dunhuangia

??

1111?0?02120?02??0??1?11?[23]2[123]?2121???200?0?10??000?????0001?????1??????

0?3110000010010120010001 2??

????????????????0?1?1021???????????2?????1??0?01101

Piscivorenantiornis

???

???

???

????????????????????21??1?1001?011??

Linyiornis

10101?02?0?????????????0?????????000???0?0??01?1??10?1?2?1?0110101??24??020?

0???0111110101[12]1?0020001?00111[23]2[123]?[12]??1??????00100??01021100???11

1011110011?0121000000100101200l0???22000??0010?0101?1100??0012?0????1?0?10

?001?110010010??0?0?0201?0011??11121021111002100?20??1?100000?101

Sulcavis

[01]010??0????????????????0?????????000???0?0?????1?????11?????11?[12]????24?10

10110??01????0?010[12]??0[01]0001?0?1113[12][123]????????????01????010????????

?1???11??[01]???0?[02]1??0[01][01]0?00?0120010101120?00?[01]?????????[12]10????

?????????0?00?0?01??110010010??0[123]??0101?0011???11211[12]011110012?0110??

1?1????01101

Bohaiornis

?0[12]01102?0?????????????0????????1000???000??0011????1?120?1?110[0123]????2

4000?01101?0111110?01020?0[01]0101?0?11122[123]?21311??30?001??100001?10?0?

011???11?????10?0110000010010120010101230000??????01011[12]??000?0?1????0?0

??0?0??10?1100100100?01?[01]0101?0?11??10121021111000121011 0??1?1000001101

Longusunguis

?0[12]01?02?0????????????????????????000???000??01?1??[12]?1??20??1110[123]????

24?00?0110??01111101020???010001?0111?32[123]????1???[23]00001??10000??10?00

011???11????10?01??0000100101?00101012200???????01011[12]??000????1?100?0?

00?00010?100?1???000?1??010100?1????112102011?1002210110?01?1000001101

Shenqiornis

[01]0101102????????0??????0?1???????000???000???1?1???11?0?01??11??????????0??

?????01?11?0?01?[12]??010101??011?[23]2[123]?[12][12]3?1??20??01101?000???????

??1???11????????01??0010100101200101011300000??????01?111???01??????????0????

????????0?1011???0???[01]?0?0?1???[12]111211[12]01??2?0?21011??01?1000001101

Zhouornis

[01]0101?01?0????????????????????0??1000???000??0??11?[12]?1??20???110?????24??

0??1?0??01111010101??020101?0111132[123]?2?3110??0?000??10000??10?00011???

11?????10?31??00001001012001010122000???????????[12]??00000??1?1?0?1?00?0?

01??110010010??01??0101?0?11??11121?2011110012?0110??1?1??0001101

Parabohaiornis

[01]0201102?0????????????????????????000???000??0??1???01??200??110[12]01?124??

0?01101101?1110?01?11?010101?0111132[123]?20311?[345]30?001??10000101010001

110111????10?01??0000100101200101012300000??0?0101011[12]1100000??????0?0?0

0?00010?100010010??01200101?0?11??1112102111100022?0110??1?1000001101

Fortunguavis

??????0????????????????????????????????00???0????0?110200??1?000??02[34]??0??1

110?0111110100111002 0????0111122??2??1???300000??10000??1??0001?1??11?????1

1221100000100101200100 0?120?????????0101111001 2?0????????1???10???1?1100100

10000?0?02?100011???11?10?1?11100111?011??1?1??0??1111

Pterygornis

00201102?0??????????????0?0?0?????000???0000001?111[12]?11??0111110211?12?000

??1101??1????010211100000 01?0?11132[123]1212100?20?00100100001110000011001

110000110?311000001001012 00???01221?0?10011??10??1100?10011?0????1?001????

1?110010011010 1?0010110?11??011?1021?1?100??000210?1?10?11011?1

Cruralispennia

[01]0?0??02????????????????0?????????000????0??????1????????0?112???????2??00???

?0[01]??1?1??01011???00?001????????2[23]12201???10000110??0001?????????????10??

0?????[23]1??00???0??0120??0???2200????????01011110003??????????1??????????110

0?00?00?????????????????1?100221???2???1????2??1?1?101??10?

Monoenantiornis

?0?0??1???????????????????0???0????000???00??????1????????0???1??1112?2???????1?0

???1??110??1??0?0000010??11??231200000????10100??0001010????1100011?????00?

????0??0100101200000??12???????????????????0000????????????1?0111??1?0?10?1???

0100?2?0?0?11??1?1?101012000012??110??1?10?0101101

Archaeorhynchus

1013111[12]????????????????1????????1?00??00100??1???01?0??0??1???1000??[34]01

11????1101011?0010?20?[01]20000?0121002[23]122211?[12]20??1000??010????0???0

110?100001100?31??000110000021010101030000??0?101100?[12]10001?0????1???1?

0[12]00????211[01]010100??0?0?0100?00????0010101100000?01[01]0020?01?10?0110

101

Schizooura

0123111[01]??????????????????????????11?0????10??01??0?[12]1?00?00?1??0[345]?1??[

12][34]?[12]0[12]?000??01011?0?1001??[01]2?00????11012[23]0[12]?011??1??1?000?

?0[12]0010[01]0???01?0?10?????00?31??0000?001?0[23]001010103?0??1[01]1???0101

?1?0103?????[12]?0?????[12]???????111010?00001[01]2?011000010?1?01?1110?10000

0010021?01?1??01?0101

Bellulornis

??1?00?[234]????2?01?110

00???1011?0?10?10?020????0?11?12[23]0[12]2211?????110000?0100100????01???100

0???00?31000000000010210100010 31???10?1??010?12100???0102?????1?02?0???1?1

12010120???02?0200?00?0??001?0011?1010?00??021??1?10?1??1101

Vorona

???

???

??????????????????????????????????????101120211?0001111011111[12]000100011?2100

0000??????????????11???????????1???????????????

Jianchangornis

??????????????????????????????????00????1?0?????1??1?????0101200[234]0??????????

?????0101110010020?0200?0?0121?02[23]031001??[01]0?0100000010010000000 1?011

1?????0???1??01?000000021010101 03??01?[01]?????????[12]?000?????2??00???0??0?

10??1120?0000??????0100?00???01010001110000?0?10?01????1??1?1100?

Songlingornis

???[01]?????????????????????????????000?????0?????1????????????2???????????????

????10?????101???2????0???21?02301231[12]??51?????????????????????????????????

???0?????????????????????????????????

?????????????1?10?01??????????????????1????1????

Longicrusavis

10[12]01?0??????????????????????????00???101??01????11?11?0???20?????????????

?????1011100?0????[12]21100?01210?23?120110?10?110?01?1101202110001000 1100?

??0??31110??00010102100010101???????11??1???[12]??0?????????01?10022[01]121?1

112011100??10100100?0011??00101010001100 00???02????1??011?101

Apsaravis

??????????????????????????1[01]?????1?1????1????????021?1?201??00?4??0?24?10??

??????10111000011111211020 01????[12]30???11?????11001000101110000111111 11012

1101?311001110110???1????????100011111?111012??113101[12]?1?0??1???20121??11

[12]?111001?1?200100?00??????1??12110??0?????0?1????1?????0??1

Hongshanornis

?020?10???????????1?????????????????00???101??0?????????????????1???????24????????0

1?0101110010????[01]211?0?0121?12??2201[12]?210?110?????10???2????0????11????

????31??00?0??00?021010101??????????0??01[12]????3???????????0?[12]???????11

20?0?00?0?0??0200?00111100101021001100001001 2?11?1??0110101

Archaeornithura

??[23]???0[12]4??111

0001?0101110010110?221100?0?2101230[12]?111???????0??10010??02????0100??????

??0??[23]1??0[12]?0?000?02100010101?0????????01001[12]???03????2??0??1?0????2

?0?1110?1?01???[01]??0100?001111?0101011?01100001???1??1?10?0??0101

Parahongshanornis

???1?1????????????????????????????

?1????0?10????[01]211???01210123022011??10??10??1?010??0?????01???11?????????3

1??01?0?00010210001010 1?0?????????10??[12]??003???????0????00???????1???11?0?

???1??0100?0011??001?1011?0110000????2????10?0?10101

Tianyuornis

1020??0??????????????????????????000????01??01?1?????????????????????4????????1

1?10???0?10????22??????1210123?22111??10?1100???010??0?????01?0?11????????[23

]1??0??0?00010210001010 1??????????????????????????????1??[12]??12???111011?01

???[01]??01?0?0?11??001?1011?0110000????2??1?1??011?101

Yanornis

10[12]11102?1????????1?????????????1000???1?00?01?1??0?????01??2003??0????????

?????01011110100?0?220001??121?02301121[12]??51011001??010110[01]????010001

1?0?11?0?31110000000010210101012310100?11?10100?11?003??1???????1?0??0??0??

111011?00[01]1112?0100?0011??0?101001000000000011?11?11?1110101

Patagopteryx

????????????????1?11110001111????????????0??000?1121101100110003 1101??120????

????1011100000?0?1210010??????2[12]????10?????1001???1000?0?00000100?000000

?0?????????0??1?1?20?00???20?0001000001110121?013?0??20????1001?0?011111201

01001110000?00?0001????10??110100000?0?0???????0????????

Yixianornis

1121???????????????????0???1?1???0?0???110?????1??1?1??2011?1003??0?24????100

0[12]00101110010010?220001001210?2301121[12]0?51??10??11??0?0?0?11001?0?100

021?0??31110101001010210101010 2100?1??00?01?111??003??1?2?201?1002???????1

1[12]01?0001110?0010??0011?110101011?0000001?0011?11??1?1?10101

Piscivoravis

???????????????????1????111?[01]????????????????????1211??10111100[34]??0?24?20

??0001001011?0??0????[23]20????0121002301121[12]??51?110??1?1?0??0??????1?0?

11?????00?31??000110001021010101121000?0????01011[12]10003?01121?01?10022??

??1?112011100[01]110?00110000101 1001?01???0000000??001?11?11?0?10101

Iteravis

00231102?0???????????????0???????000???1?0??01?1??[12]1????01?12?0[123]???024?

20110001101011?0?10?10?220????0121002[23]01111[01]??51?11000??01?1?02????01?

??11?????0113121010100011021010101 22100?11111?01011110000?0102120??100[12]

?1?00??11211110???100?0110?0011??101?101?010100000022?11?11?0110101

Gansus

???121?1?201111004001024?2021000

0?1101110010020?320???001210023021010 0?10?11001??010130?1???0100111001 11?0

?312?01110001?131010001221?001111010101121000 3001[12]?1201?100[12]2?0?0?21

1201110010102?020000011??001?10110?010000??023?11?11?1?10101

Ichthyornis

21[12]???0?????????111111??1111???01001???10021011111[01]11012011120?[34]?010

2401021000???101111010010?2210110012?002300??1212?011100110010120210?1010

0011002110 1?3121012101001031111????310?01011??1101121?11?101221201?100221

110?2112011202?01?210200000??????10101001?1????0?????11?10??100101

Hesperornis

11231103?1021011?111111000111?1?10100??100010101?0210012111120 04010?13020

2100010?0?01?00?0010?020???000200?21?0??0011?00??????0?????1000000?????????

?????????????????????????????10001211011100121011310212220111012211111211201

12?21013223220102??????0??01?020?0?0?0???3?11?00??1?????

Parahesperornsi

1123??0???0?10??1?111???00111????01????1???????1?02100121111200?????1?????10

001??0?01?0??0010?0????????????2???????1?????????????????????????????????????

???10212220111012211 11???1201?2?2101?

223220002?????????????20?0?0?????3????1?????????

Enaliornis

??????????????????????????11??????????????????????21?0??1???2???????????????????

???

????????????????1??????1???????2?????1012212011101221111???1201???1101???0120

000????????????02?????????????????????????

Baptornis_advenus

1??3??????????????11???00?111???????0??1?0?10101?021001?11112004010?13020?10

0011?0001?0010010?0?????0??????2[12]????0112??0????????????1000000????000001

00?????????????????????????01000121101110012101131011212011101221111211201

??2?11013221120001????????????02??????????3????00????????

Baptornis_varneri

???021?01?1??1????????????????????

???

????????????????10????1???1100?210113?????2201?101221111211201?2?1101?22122

0001?????????????2?????????????????????????

Vegavis

??2????????????[56]??????????????

???10111001001?0[01]20100?01?????????????????1100111???0???????????????????01

2?1??022110010031111??12301???1?????01??2??????01121201?1002?2?1????12011??

?3?1????1???0???????101011???????????23????1?????00?1

Anas

21230113101111111111111111112112111?102100001011?10211012111100060120240202

1000201101111010020?22010000121002311100113401110011110003011111010000100

1110123120132111111031011??123110111101011112101131011212011100222011121

1201120130132102200001011101011210101000100023?11?10?1100???

Gallus

11231113111211111111111111112112111?102000001011?10211012111100160120240202

1000201121111010020?22110000121012311010112201110011110003011111110001100

1110113120132102101131011??1211101121101101112101131011212011100222011121

1201120130132201201001011101011210101000100022?11?10?1100???

Yangavis

[12]0231112????????0????????????????1?0???01001100???10?1?[01]0??10?01???0??0?

0?????110??010??00????020?0??0000002[12]?0??0???10?000000?02100000???01???01

?00?00??210?0000100000000002100300???????????111??003?0?1??????1??0?0000?0?

10000100??0?2?0100?0?11???00?1??00100000100000??1?0000101010

Jinguofortis

???0??0??????????????????????????000???0?00001?1??[12]0?0?2??010?01????24??020

??00?0??0??0????????00????01000021????00????0001001?0100?00?0??0100111??0?00

0?21??000110100010010101130000??0?0?0101111000[01]?01??01?0???00????????100

?000?000??11200?0?12?0?00?1??0?211000??0000??1?0000?10101

IVPPV25033

20[12]0??0?????????????????????????????????00?????1????1??????????2??0?24?00201

10??01111101020???0[01]0001?0011122[123]?2?310???00001??10000??00?00011???11
??0?11??21??0000100001200100011200000??0?0101011[12]1010000????100?1???10?00
??1100100110002?0000110011??01111111?1010021?0021??1??001100101

Fukuipteryx

??0???0?0?00?0011200?????2??202?000
??01011?01000?0?0????????000??????????????1000000?1?0010000000101000001000??
??0????110???0?0??0??0?3?0?00000?1???????????0010110100?001??????????0????000
???0?0???0???0??????000???0????0???0?????????0?00?

Supplementary References

1. Shibata, M., & Goto, M. Report of the 3rd Dinosaur Excavation Project in Katusyama, Fukui, 2007. *Mem. Fukui Prefect. Dinosaur Museum* **7,** 109–116 (2008).
2. Wang, M., Stidham, T. A. & Zhou, Z. A new clade of basal Early Cretaceous pygostylian birds and developmental plasticity of the avian shoulder girdle. *Proc. Natl. Acad. Sci.* 201812176 (2018). doi:10.1073/pnas.1812176115
3. Mayr, G., Pohl, B., Hartman, S. & Peters, D. S. The tenth skeletal specimen of *Archaeopteryx*. Zool. J. Linn. Soc. **149,** 97–116 (2007).
4. Wang, M., Wang, X., Wang, Y. & Zhou, Z. A new basal bird from China with implications for morphological diversity in early birds. *Sci. Rep.* **6,** 1–12 (2016).
5. Chiappe, L. M., Shu'an, J., Qiang, J. & Norell, M. Anatomy and Systematics of the

Confuciusornithidae (Theropoda: Aves) from the Late Mesozoic of Northeastern China. *Bull. Am. Museum Nat. Hist.* **242,** 89 (1999).

6. Zhou, Z. & Zhang, F. Anatomy of the primitive bird *Sapeornis chaoyangensis* from the Early Cretaceous of Liaoning, China. *Can. J. Earth Sci.* **40,** 731–747 (2003).

7. Sano, S. New view of the stratigraphy of the Tetori Group in Central Japan. *Mem. Fukui Prefect. Dinosaur Museum* **14,** 25–61 (2015).

8. Amiot, R. *et al.* Environment and ecology of East Asian dinosaurs during the Early Cretaceous inferred from stable oxygen and carbon isotopes in apatite. *J. Asian Earth Sci.* **98,** 358–370 (2015).

9. Sano, S. & Yabe, A. Fauna and flora of Early Cretaceous Tetori Group in Central Japan: The clues to revealing the evolution of Cretaceous terrestrial ecosystem in East Asia. *Palaeoworld* **26,** 253–267 (2017).

10. Suzuki, S. *et al.* Sedimentary environment of dinosaur fossil bearing successions of the Lower Cretaceous Kitadani Formation, Tetori Group, Katsuyama City, Fukui, Japan. *Mem. Fukui Prefect. Dinosaur Museum* **2015,** 1–9 (2015).

11. Rauhut, O. W. M. New observations on the skull of Archaeopteryx. Paläontol Z **88,** 211–221. (2014).

12. Lefèvre, U. , Hu, D. , Escuillié, F. , Dyke, G. & Godefroit, P. A. A New Long-tailed Basal bird. Biol. J. Linn. Soc. Lond. **113,** 790–804. (2014).

13. O'Connor J. K., Chiappe, L. M., Gao, C., & Zhao, B. Anatomy of the Early

Cretaceous Enantiornithine Bird *Rapaxavis pani*. Acta Palaeontol. Pol. **56,** 463–475. (2011).

14. Field, D. J. *et al.* Complete Ichthyornis skull illuminates mosaic assembly of the avian head. Nature **557,** 96–100 (2018).
