# Supplementary material for: An unusual bird (Theropoda, Avialae) from the Early Cretaceous of Japan suggests complex evolutionary history of basal birds
Source: Commun Biol. 2019 Nov 14;2:399. doi: 10.1038/s42003-019-0639-4 (PMC6856171; doi:10.1038/s42003-019-0639-4)
Supplement: Supplementary file 2 — Reporting Summary [file 42003_2019_639_MOESM2_ESM.pdf]

nature research

Corresponding author(s): Takuya Imai

Last updated by author(s): Sep 15, 2019

Reporting Summary

Nature Research wishes to improve the reproducibility of the work that we publish. This form provides structure for consistency and transparency in reporting. For further information on Nature Research policies, see Authors & Referees and the Editorial Policy Checklist.

Statistics

For all statistical analyses, confirm that the following items are present in the figure legend, table legend, main text, or Methods section.

n/a	Confirmed	
☒	☐	The exact sample size (*n*) for each experimental group/condition, given as a discrete number and unit of measurement
☒	☐	A statement on whether measurements were taken from distinct samples or whether the same sample was measured repeatedly
☒	☐	The statistical test(s) used AND whether they are one- or two-sided<br>*Only common tests should be described solely by name; describe more complex techniques in the Methods section.*
☒	☐	A description of all covariates tested
☒	☐	A description of any assumptions or corrections, such as tests of normality and adjustment for multiple comparisons
☒	☐	A full description of the statistical parameters including central tendency (e.g. means) or other basic estimates (e.g. regression coefficient) AND variation (e.g. standard deviation) or associated estimates of uncertainty (e.g. confidence intervals)
☒	☐	For null hypothesis testing, the test statistic (e.g. *F*, *t*, *r*) with confidence intervals, effect sizes, degrees of freedom and *P* value noted<br>*Give P values as exact values whenever suitable.*
☒	☐	For Bayesian analysis, information on the choice of priors and Markov chain Monte Carlo settings
☒	☐	For hierarchical and complex designs, identification of the appropriate level for tests and full reporting of outcomes
☒	☐	Estimates of effect sizes (e.g. Cohen's *d*, Pearson's *r*), indicating how they were calculated

Our web collection on statistics for biologists contains articles on many of the points above.

Software and code

Policy information about availability of computer code

Data collection	No software was used.
Data analysis	We used TNT v. 1.5^49 for phylogenetic analyses.<br>The description and character coding for the software are provided in the method and supplementary data of the manuscript.

For manuscripts utilizing custom algorithms or software that are central to the research but not yet described in published literature, software must be made available to editors/reviewers. We strongly encourage code deposition in a community repository (e.g. GitHub). See the Nature Research guidelines for submitting code & software for further information.

Data

Policy information about availability of data

All manuscripts must include a data availability statement. This statement should provide the following information, where applicable:

- Accession codes, unique identifiers, or web links for publicly available datasets
- A list of figures that have associated raw data
- A description of any restrictions on data availability

The specimen (FPDM-V-9769) is housed in Fukui Prefectural Dinosaur Museum, Katsuyama, Fukui, Japan. Morphological character descriptions and character datasets for phylogenetic analyses are included as Supplementary Data. The rendered three-dimensional model for FPDM-V-9769 is available at 10.6084/m9.figshare.9869537.

Field-specific reporting

Please select the one below that is the best fit for your research. If you are not sure, read the appropriate sections before making your selection.

☐ Life sciences ☐ Behavioural & social sciences ☒ Ecological, evolutionary & environmental sciences

For a reference copy of the document with all sections, see nature.com/documents/nr-reporting-summary-flat.pdf

Ecological, evolutionary & environmental sciences study design

All studies must disclose on these points even when the disclosure is negative.

Study description	An associated skeleton of fossil bird, FPDM-V-9769, collected in Fukui, central Japan is described and compared with other fossil birds. Its phylogenetic position is hypothesized.
Research sample	FPDM-V-9769, an associated skeleton of a fossil bird came from the Kitadani Formation, Katsuyama, Fukui, Japan. For phylogenetic analyses, we used the dataset for character matrix of fossil and extant birds provided in Wang et al., 2018 (see the method in the manuscript).
Sampling strategy	We did not perform any sampling.
Data collection	Takuya Imai, Yoichi Azuma, Min Wang, and Zhonghe Zhou collected morphological data of the specimen through direct observation on specimens and CT images. The method for the collection of CT images is detailed in the manuscript.
Timing and spatial scale	Our study did not involve periodical sampling.
Data exclusions	No data were excluded from the study.
Reproducibility	We did not take measures to verify the reproducibility because the specimen description did not involve experiments. The procedure for CT experiments to obtain CT slice images is provided in the manuscript.
Randomization	This is not relevant to our study because the study did not involve any grouping.
Blinding	Blinding was not relevant to our study because the study is conducted based on a single specimen.

Did the study involve field work? ☐ Yes ☒ No

Reporting for specific materials, systems and methods

We require information from authors about some types of materials, experimental systems and methods used in many studies. Here, indicate whether each material, system or method listed is relevant to your study. If you are not sure if a list item applies to your research, read the appropriate section before selecting a response.

Materials & experimental systems

n/a	Involved in the study	
☒	☐	Antibodies
☒	☐	Eukaryotic cell lines
☐	☒	Palaeontology
☒	☐	Animals and other organisms
☒	☐	Human research participants
☒	☐	Clinical data

Methods

n/a	Involved in the study	
☒	☐	ChIP-seq
☒	☐	Flow cytometry
☒	☐	MRI-based neuroimaging

Palaeontology

Specimen provenance	The specimen was collected from the Kitadani Dinosaur Quarry (36.122, 136.544), Katsuyama, Fukui, Japan. The quarry is owned by Katsuyama City Government, which issued a permit t Fukui Prefectural Dinosaur Museum to conduct excavation and collect fossil specimens.
Specimen deposition	The specimen is deposited at Fukui Prefectural Dinosaur Museum, Katsuyama, Fukui, Japan.
Dating methods	No new dates are provided.

☐ Tick this box to confirm that the raw and calibrated dates are available in the paper or in Supplementary Information.
